# Far-red timing uncovers cultivar-dependent yield and bolting responses in vertical-farm spinach (*Spinacia oleracea* L.)

**DOI:** 10.3389/fpls.2026.1934445

**Published:** 2026-10-13

**Authors:** Christopher McGovern, Milena Adrio, Hadi Aliki, Ruth Vichos, Wayne Powell, Rajiv Sharma

**Affiliations:** Horticultural Innovation Centre, School of Natural and Social Sciences, Scotland’s Rural College (SRUC), Edinburgh, United Kingdom

**Keywords:** bolting, controlled-environment agriculture, far-red light, genotype × environment × management, light recipe, shade avoidance, *Spinacia oleracea*, vertical farming

## Abstract

Far-red light (FR; 700–750 nm) is increasingly incorporated into controlled-environment lighting because it can improve photosynthetic efficiency when combined with shorter photosynthetically active wavelengths, principally red and blue. In long-day leafy crops such as spinach, however, FR may also promote the transition from vegetative to reproductive growth and thereby reduce marketable yield. Most studies have evaluated FR fraction, intensity, or end-of-day exposure, whereas the developmental timing of FR has rarely been tested, particularly in spinach. Here we evaluated six commercial spinach cultivars (Amador, Harp, Renegade, Responder, Rubino, and Santa Cruz) in an indoor vertical farm under a common red–green–blue background (PPFD 260–264 µmol m^–2^ s^–1^, 12-h photoperiod, 24 °C) and four FR timing treatments: no FR (control), FR throughout production (FullFR), FR during early development only (EarlyFR), and FR during late development only (LateFR). Far-red was supplied additively on the constant PAR background (i.e., supplemented, not substituted for PAR photons). LateFR matched or slightly exceeded control in marketable fresh weight (244 vs. 224 g; not significant) without increasing flowering incidence whereas far-red supplied during early development reduced fresh weight (158 g) and increased flowering. The magnitude of the timing response differed among cultivars: switching from EarlyFR to LateFR recovered 0% fresh weight in Amador but 106–107% in Renegade and Rubino, with the largest penalties occurring in otherwise bolt-resistant cultivars. EarlyFR also increased total chlorophyll and reduced the chlorophyll a/b ratio. These results show that FR response in spinach is strongly conditioned by developmental stage and cultivar. Although LateFR received more total far-red than EarlyFR, it behaved like the control, indicating that the penalty was set by far-red timing rather than dose. Treatment differences in bolting and yield tracked an estimated phytochrome photostationary-state deficit during early development: a phytochrome-deficit model markedly outperformed a cumulative-dose model (ΔAIC = 33), and the deficit × cultivar interaction was strong (*p* < 0.001), with bolt-resistant cultivars losing most yield when far-red coincided with the early developmental window. We therefore propose that FR should be treated as a genotype-dependent management variable rather than as a fixed spectral input, with late application and bolt-resistant cultivars offering the most favorable combination for vertical-farm spinach production. Framed within the breeder’s equation, the close match between the trial and production environment and the scope for shorter breeding cycles indoors suggest that genotype and far-red timing can be optimized jointly to accelerate genetic gain.

## Introduction

In northern Europe and other high-latitude regions, fresh vegetable supply is increasingly affected by population growth, urbanization, climate instability, and disruption to long food, fertilizer, and energy supply chains. These pressures have increased interest in local, season-independent production systems that can buffer seasonal shortages and reduce dependence on long-distance transport (Girardet, 2008; Sulaiman and Mirabi, 2023). Leafy vegetables are an important target because field production is highly seasonal and because warm, long-day conditions can accelerate flowering in cool-season crops.

Controlled-environment vertical farms (CEVFs) decouple crop production from season and latitude by replacing sunlight with light-emitting diodes (LEDs) and maintaining defined set-points for temperature, humidity, CO_2_, and nutrition (Martínez-Moreno et al., 2024; Farhangi et al., 2025). These systems can increase land-use efficiency, shorten supply chains, and reduce pesticide requirements by confining nutrient delivery to a closed recirculating system (Despommier, 2011; Sulaiman and Mirabi, 2023). Whether inputs are lower than in field production depends on the comparison drawn, and the advantage is smaller relative to organic systems. The principal constraint on indoor vertical farming remains the high operational cost, particularly electricity for artificial lighting. Rising energy prices in recent years, driven by the COVID-19 pandemic and subsequent disruptions to global energy markets, have further increased production costs (Ferrarezi et al., 2024). Consequently, optimization of indoor leafy-green production requires light recipes that improve marketable output per unit energy rather than simply maximize biomass.

Spinach (*Spinacia oleracea* L.) is well suited to vertical production in several respects: it is compact, fast-growing, nutritionally dense, and increasingly demanded in urban diets (Zou et al., 2020; Martínez-Moreno et al., 2024). It is also agronomically challenging because it is a long-day crop that bolts readily under photoperiods of approximately 13–15 h or under warm conditions. Bolting shifts growth from harvestable rosettes to reproductive stem elongation, reducing yield and quality traits (Chun et al., 2000; Semenova et al., 2023; Amini et al., 2025). Managing the balance between vegetative growth and reproductive transition is, therefore, central to indoor spinach production.

For plants, the red (R; 600–700 nm) to far-red (FR; 700–750 nm) ratio is a major signal of neighboring vegetation. Chlorophyll absorption reduces R relative to FR within dense canopies, and plants interpret a low R/FR ratio as an indication of competition (Li et al., 2011; Wei et al., 2023). This signal is perceived and transduced mainly through phytochrome B and PHYTOCHROME-INTERACTING FACTOR pathways and can induce shade-avoidance responses, including stem and petiole elongation, hyponasty, altered chlorophyll partitioning, and earlier flowering or bolting (Franklin and Whitelam, 2005; Casal, 2012; Paik and Huq, 2019). Depending on genotype, developmental stage, and abiotic context, notably air temperature, a low R/FR can also elicit shade-tolerance responses such as leaf-area expansion rather than elongation (Gommers et al., 2013; Halliday et al., 2003). At the same time, FR photons can contribute to whole-canopy photosynthesis when combined with shorter wavelengths by re-balancing excitation between photosystems I and II (Zhen and Bugbee, 2020); this enhancement is not universal, however, and depends on the shorter-wavelength background and on temperature, with some studies reporting little photosynthetic gain and predominantly morphological responses to supplemental FR (Jeong et al., 2024). Thus, FR may increase light-use efficiency but can also activate developmental responses that reduce spinach marketability and thus profitability.

A low R/FR ratio also accelerates the floral transition in many long-day species, acting through phytochrome B on the FLOWERING LOCUS T pathway, and supplemental far-red has been shown to advance flowering in lettuce, *Arabidopsis*, and several ornamental species (Craig and Runkle, 2016; Park and Runkle, 2019; Kalaitzoglou et al., 2019). In leafy crops harvested vegetatively, this response is a direct commercial liability rather than a benefit.

Most work on FR in controlled environments has used model species or lettuce and has applied FR continuously or as an end-of-day treatment (Li and Kubota, 2009; Kalaitzoglou et al., 2019; Tan et al., 2022). Spinach has been studied less extensively, and existing research has focused primarily on light quality (far-red fraction) and light quantity (photon flux density), while the influence of light duration (photoperiod) and the temporal distribution of far-red light within the photoperiod has received little attention (Utasi et al., 2019; Skabelund et al., 2025). In lettuce, far-red applied during early growth was recently reported to increase biomass (Levine et al., 2026), although whether early far-red is beneficial or detrimental appears to depend on the species and, within a species, on genotype and its bolting behavior. Skabelund et al. (2025) reported no morphological response of spinach to increasing far-red fraction across a wide range of photon fluxes, contrasting with responses reported in other leafy crops.

In this study, we tested FR timing as a management factor and evaluated its interaction with cultivar within a genotype × environment × management (G × E × M) framework. The objective was to determine how the timing of supplemental FR affects yield, leaf morphology, chlorophyll traits, and bolting in spinach and to identify whether cultivars differ in their sensitivity to FR timing. We hypothesized that FR would increase flowering probability and plant height, increase yield when applied at an appropriate stage, have a neutral or negative effect on chlorophyll content, and produce cultivar-dependent responses.

## Materials and methods

### Plant material and cultivation

Six commercial spinach cultivars (Amador, Harp, Renegade, Responder, Rubino, and Santa Cruz; Moles Seeds Ltd., UK) were sown in QuickPot QP D 40/4.5 R propagation trays (HerkuPlast Kubern GmbH, Ering/Inn, Germany; 530 × 310 mm, 40 cells, 45 mm deep, 57 cm^3^ per cell) containing a 90:10 (v/v) coco-coir/vermiculite substrate, identical for every cultivar and light treatment. These six cultivars represent the commercial spinach material currently available to UK vertical-farm growers and were chosen to span a broad range of reported bolting behavior and leaf morphotype, from bolt-prone to comparatively bolt-resistant types, so that genotype-dependent responses to far-red timing could be resolved. Bolting-resistance scores and observed flowering incidence for each cultivar are given in **Supplementary Table S4**.

All cultivars were sown on the same day with no staggering. The trays were placed in an Intelligent Growth Solutions (IGS) Growth Tower (model GT6; Intelligent Growth Solutions Ltd., Dundee, Scotland, UK) and germinated in darkness for 7 days. The trays were transferred to the light treatments only once emergence was judged uniform across all trays and all cultivars, assessed visually at transfer; no formal germination assay was carried out. The number of plants scored in each tray at harvest is given in the deposited tray-level data, so any systematic difference in establishment among cultivars or treatments can be checked directly.

At 2 weeks after emergence, a nematode drench (Nemasys Biological Fruit and Veg Protection) and sticky traps were applied for sciarid fly control. Nutrient solution (90 L; composition shown in **Table 1**) was supplied by ebb-and-flow every 2 to 3 days at pH 5.8 and electrical conductivity 2.0 mS cm^–1^. Air temperature, relative humidity, CO_2_ concentration, and photoperiod were regulated by the Growth Tower’s integrated climate and CO_2_ control; set points and logged values are given under “Experimental design”.

**Table 1:** Nutrient solution composition (per 10 L of stock).

Stock	Component	g per 10 L
A	Calcium nitrate	960
A	Potassium nitrate	28
A	Iron EDDHA 6%	75
B	Potassium nitrate	462
B	Magnesium sulphate	350
B	Monopotassium phosphate	160
B	Magnesium nitrate	60
B	Manganese sulphate	3.6
B	Disodium octaborate (21% B)	2.4
B	Zinc sulphate	1.5
B	Copper sulphate	0.4
B	Sodium molybdate	0.2

### Light treatments and timing

All treatments received the same red–green–blue background spectrum at a mean PPFD of 260–264 µmol m^–2^ s^–1^ and an approximate daily light integral of 11.3 mol m^–2^ day^–1^ over 12 h. FR was supplied additively at 26 µmol m^–2^ s^–1^, equivalent to 10% of PPFD, and was applied coincident with the 12-h photoperiod throughout the far-red window rather than as an end-of-day or night-interruption treatment. Four timing treatments were imposed: control (no FR), FullFR (FR throughout the 32-day cycle), EarlyFR (FR during days 0–12 only), and LateFR (FR from the early/mid transition to harvest). Because FR was added to a constant PAR background, total photon flux differed between FR and non-FR phases, while PAR remained constant. FR is, therefore, interpreted as a supplemental signal rather than a replacement for PAR. We supplemented far-red on a fixed PAR background rather than substituting far-red for PAR photons: substitution designs hold total photon flux constant and are appropriate for testing the photosynthetic equivalence of far-red (Zhen and Bugbee, 2020; Skabelund et al., 2025), whereas our question concerned the photomorphogenic signal carried by far-red timing. Supplementation isolates the phytochrome signal at a constant, horticulturally realistic PAR level. The design introduces a small difference in total photon flux between far-red and non-far-red phases, which we address directly (see below): LateFR carried a larger cumulative far-red dose than EarlyFR yet patterned with the control, and a phytochrome-state model markedly outperformed a cumulative-dose model, so the timing effects are attributable to phytochrome signaling rather than to the small accompanying increase in photosynthetic photons. The treatment recipes are summarized in **Table 2**. PAR was provided by the Growth Tower’s integrated multi-channel LED array, with independently programmable and dimmable white, red (~660 nm), green, and blue channels and supplemental far-red (~731 nm) by a dedicated far-red channel; the per-phase channel outputs, together with PPFD, total photon flux density, computed yield photon flux density (YPFD), and daily light integral, are given in **Supplementary Table S7**. To test whether the timing effect reflected phytochrome state rather than far-red dose, we estimated the phytochrome photostationary state (PSS, *φ* = Pfr/Ptot) for each treatment from the programmed channel photon fluxes (red ~660 nm, far-red ~731 nm, with minor blue and green contributions), following *φ* = Σ Nλ σrλ/Σ Nλ (σrλ + σfrλ) (Sager et al., 1988; **Supplementary Tables S1–S3**).

**Table 2:** Far-red timing treatments.

Treatment	Far-red window	Days with FR	FR (µmol m^–2^ s^–1^)	Total cycle (days)
Control	None—RGB background only	—	0	32
FullFR	Whole cycle	0–32	26	32
EarlyFR	Early development only	0–12	26	32
LateFR	Late development only	13–31	26	32

All treatments received the same red–green–blue background (PPFD 260–264 µmol m^–2^ s^–1^; 12-h photoperiod; DLI approximately 11.2–11.4 mol m^–2^ day^–1^). Far-red, where applied, was supplied additively at 26 µmol m^–2^ s^–1^ (10% of PPFD).

The delivered photosynthetic photon flux density was verified independently after the growing period using two separately calibrated instruments: a Skye SKP215 quantum sensor (400–700 nm, ± 5%) on a 10 × 5 grid spanning the growing area of each growth tray (*n* = 50 per recipe) and a LI-COR LI-180 spectrometer (380–780 nm, ± 5%) at central and lateral positions in three of the four recipes. Measurements were made at approximately 150 mm below the LED plane, corresponding to canopy height, with the tower running the programmed experimental recipes. The recipe means were 330.8, 315.3, 333.0, and 334.8 µmol m^–2^ s^–1^ for control, FullFR, EarlyFR, and LateFR respectively, a total spread of 5.9%, with coefficients of variation across the growing area of 7.5%–11.3% and consistently lower values toward the tray ends and outer columns (**Supplementary Figures S2**, **S3**). Agreement between the two instruments was 5.0% for control and 3.6% for FullFR, but 15.3% for LateFR, where the LI-180 set was taken at lateral positions only on a single occasion and included one clearly anomalous reading (192.5 µmol m^–2^ s^–1^, against a next-lowest value of 272.7). The Skye grid, which samples the whole growing area systematically, is therefore treated as the primary record. The conclusion does not depend on which instrument is used: under the Skye ordering LateFR is the brightest recipe, and under the LI-180 ordering it is the dimmest, yet in both cases the recipes that group together phenotypically do not group together on measured PPFD (**Supplementary Figure S3**, **Supplementary Table S11**).

### Experimental design

Each of the four far-red timing treatments was applied to one growth tray within a single IGS growth tower. All four growth trays were operated concurrently from a single sowing date under otherwise identical set points, with a common nutrient solution and irrigation schedule. The climate set points were 24 °C, 60% relative humidity, and 800 ppm CO_2_ with a 12-h photoperiod. Conditions were logged by the tower management system every 30 s throughout the experiment; the logged means were 23.0 °C, 54.5% relative humidity, and 748 ppm CO_2_ (**Supplementary Figure S4**). A single sensor serves the tower, so these records describe the shared microclimate rather than individual growth trays; because all four treatments ran concurrently, the logged variation was common to all of them.

Within each growth tray, 30 propagation trays were arranged in a 6 × 5 grid comprising six cultivars with five replicate propagation trays per cultivar (*n* = 5 trays per cultivar per treatment; *n* = 30 trays per treatment). The cultivar positions were randomized once, and the same randomized layout was used in all four growth trays so that each cultivar occupied the same set of positions in every treatment. Each of the five grid columns contained all six cultivars exactly once and was therefore treated as a complete block. Each propagation tray contained approximately 40 plants, which were treated as sub-samples. The full layout is shown in **Supplementary Figure S5** and the cultivar-to-position assignment in **Supplementary Table S8**. Because spectrally distinct light recipes cannot be applied within a single illuminated growing space, the timing treatments could not be replicated across growth trays; the consequences for inference are set out below and in the “Discussion”.

### Data collection

Plant height, leaf length (LL), and leaf width (LW) were measured on five randomly selected plants per tray at 17 days (T1) and 32 days (T2) after emergence, that is, 24 and 39 days after sowing, the light treatments having begun at emergence. No measurement was taken at the start of the light treatments; the T1 data serve this purpose in part since treatments did not differ within cultivars at that point, except in Rubino (**Figure 1**). Height was measured from the substrate surface to the newest apical growth; at T2, where bolting occurred, height was measured to the topmost growth or flower. This measure, therefore, partly includes bolting and is interpreted accordingly in the “Discussion”. Aerial fresh weight was recorded immediately after harvest, and samples were then oven-dried at 80 °C for 72 h to determine dry weight. Chlorophyll was extracted from random two leaf samples per tray by boiling in at least 99% ethanol at 85 °C for 15 min, followed by absorbance readings at 647 and 664 nm; chlorophyll concentrations were calculated using the ethanol-based equations of Ritchie (2006). Total chlorophyll and chlorophyll a/b ratios were calculated per sample and are reported as relative values rather than per unit leaf mass. Flowering incidence was recorded as the number of flowering or bolting plants divided by the total number of plants per tray at harvest.

**Figure 1:**
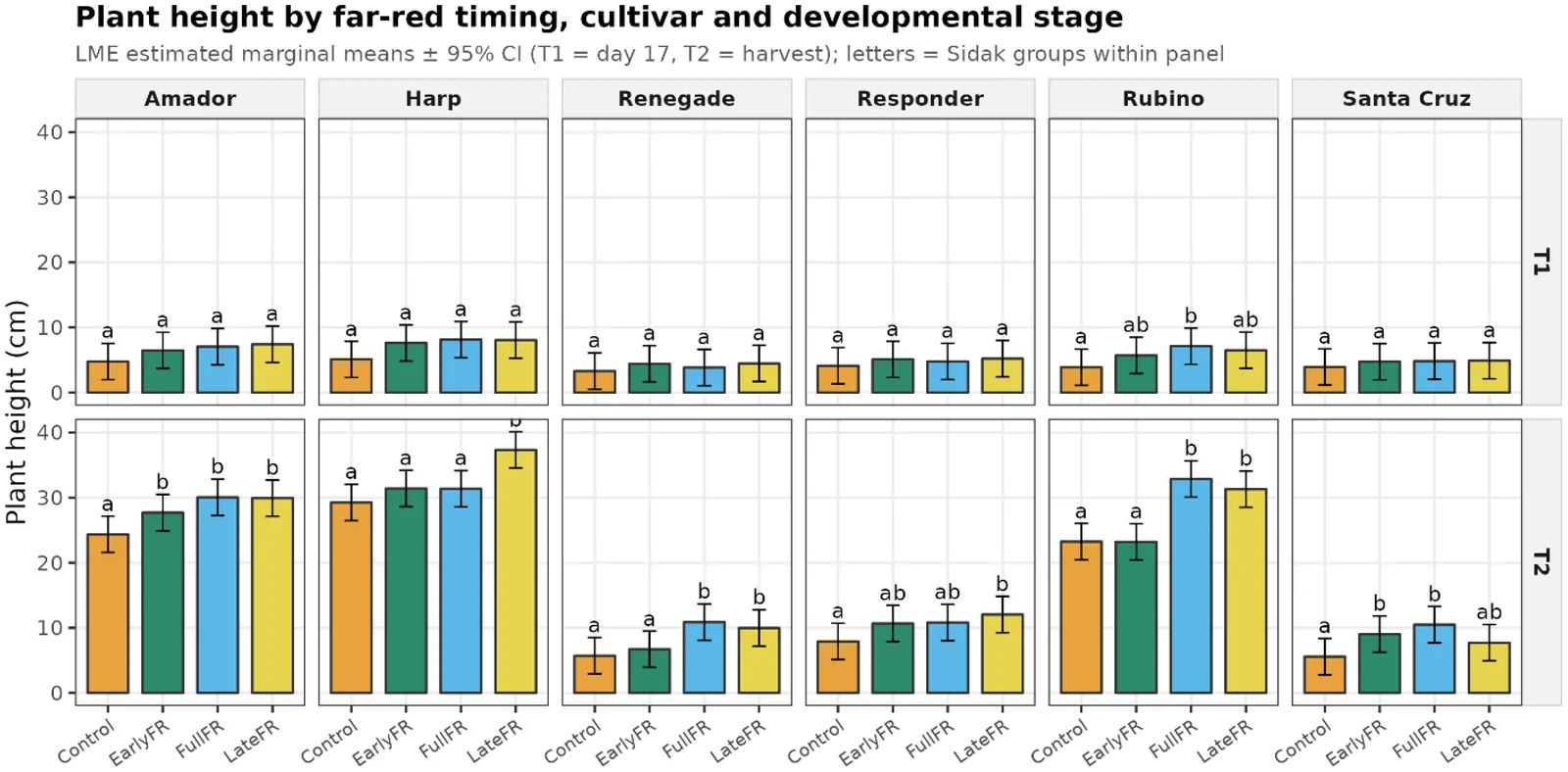
Plant height by far-red timing, cultivar, and developmental stage (T1 = day 17, upper row; T2 = harvest, lower row). Bars represent LME estimated marginal means ± 95% CI; letters indicate Sidak-adjusted groups within each panel. Treatment differences were negligible at T1 but evident by T2.

### Statistical analysis

Analyses were conducted in R version 4.6.1 (R Core Team, 2026; R Foundation for Statistical Computing, Vienna, Austria). Continuous traits were analyzed using linear mixed-effects models (using lme4::lmer function) with treatment and cultivar as fixed effects. All traits were analyzed at tray level. Plant-level measurements of height and leaf dimensions were averaged to tray means before analysis because the five plants measured per propagation tray are sub-samples of one experimental unit rather than independent replicates; the intraclass correlation among plants within trays was 0.32 for leaf length, 0.25 for leaf width, and 0.45 for height. The cultivar-by-block combination was fitted as the whole-plot stratum so that cultivar was tested against variation among whole plots and treatment and the treatment × cultivar interaction against variation within them. Sum-to-zero contrasts were used so that type III Wald *F* tests (car::Anova with Kenward–Roger denominator degrees of freedom) tested main effects in the presence of interactions. Height was analyzed across both sampling times using a time × treatment × cultivar model. Significant effects were followed by estimated marginal means (emmeans) and Tukey-adjusted pairwise comparisons or Sidak-adjusted comparisons for plant height and the bolting model. Bolting incidence was analyzed as a beta-binomial generalized linear mixed model (glmmTMB) with block as a random effect because a binomial model was overdispersed by approximately ninefold; the treatment × cultivar interaction was tested using likelihood-ratio test. One cell (Harp under LateFR) reached 100% bolting, and its estimate is, therefore, at the parameter boundary. The comparison of the two competing treatment-level predictors (**Supplementary Table S5**) was fitted on the same 120 tray-level records, using a beta-binomial GLMM for bolting and a maximum-likelihood linear mixed model for fresh weight, each with block as a random effect.

Fresh weight was also analyzed with flowering proportion included as a covariate. Significance was assessed at *p* < 0.05. Figures, compact-letter displays, and the consolidated model summary (**Table 3**) were generated from reproducible R and Python scripts applied to the master dataset. Variance components for treatment, cultivar, their interaction, block, and residual were estimated using restricted maximum likelihood and expressed as percentages of total variance (**Table 4**). The components were estimated at the boundary for both chlorophyll traits, so stratum-based fixed-effect tests are reported throughout. A quantitative-genetic summary of the phenotypic variation observed across cultivars, including entry-mean repeatability estimates with bootstrap confidence intervals, is provided in **Supplementary Text S1**; **Supplementary Table S6**.

**Table 3:** Consolidated summary of fixed-effect tests.

Response	Effect	*F*/*χ*^2^	df	df (resid.)	*p*
Fresh weight (g)	T	9.42	3	72	<0.001
	V	8.56	5	24	<0.001
	T × V	1.37	15	72	0.184
Dry weight (g)	T	5.06	3	72	0.003
	V	13.7	5	24	<0.001
	T × V	1.86	15	72	0.043
Leaf length T2 (cm)	T	20.42	3	72	<0.001
	V	16.54	5	24	<0.001
	T × V	1.52	15	72	0.121
Leaf width T2 (cm)	T	17.87	3	72	<0.001
	V	25.71	5	24	<0.001
	T × V	1.78	15	72	0.054
Leaf length/width T2	T	9.06	3	72	<0.001
	V	100.99	5	24	<0.001
	T × V	1.31	15	72	0.219
Total chlorophyll	T	17.29	3	72	<0.001
	V	0.62	5	24	0.684
	T × V	1.13	15	72	0.348
Chlorophyll a/b ratio	T	16.85	3	72	<0.001
	V	0.75	5	24	0.591
	T × V	0.71	15	72	0.769
Plant height (cm)	Time	1,042.19	1	96	<0.001
	T	16.77	3	72	<0.001
	V	68.82	5	24	<0.001
	Time × T	4.46	3	96	0.006
	Time × V	95.71	5	96	<0.001
	T × V	1.26	15	72	0.25
	Time × T × V	0.81	15	96	0.659
Flowering-adj. fresh weight (g)	Flowering (cov.)	5.6	1	93.6	0.02
	T	11.8	3	75.2	<0.001
	V	1.73	5	28	0.16
	T × V	1.43	15	71.6	0.158
Bolting (beta-binomial GLMM, *χ*^2^)	T × V	38.23	15	—	<0.001

All traits were analyzed at tray level; values are type III *F* statistics from split-plot models with the cultivar-by-block combination as the whole-plot stratum, except for bolting, where the row reports the beta-binomial GLMM likelihood-ratio *χ*^2^. The flowering-adjusted fresh weight model was fitted as a linear mixed model with Kenward–Roger denominator degrees of freedom. Plant height was fitted across both sampling times, so its time terms are tested within trays. T, far-red timing treatment; V, cultivar.

**Table 4:** Variance components as percentages of total variance.

Trait	Treatment	Cultivar	T × V	Block	Residual
Marketable fresh weight (g)	15.3	21.9	4.9	4.0	53.9
Dry weight (g)	5.3	34.3	8.8	3.0	48.6
Leaf length T2 (cm)	21.6	36.4	4.4	7.5	30.0
Leaf width T2 (cm)	9.5	62.5	2.2	5.2	20.6
Leaf length/width T2	5.1	75.0	1.4	0.0	18.5
Plant height T2 (cm)	3.7	81.1	0.3	3.5	11.3
Total chlorophyll	33.8	0.0	0.0	0.0	66.2
Chlorophyll a/b ratio	35.1	0.0	0.0	0.0	64.9
Flowering incidence	10.0	62.2	2.1	4.2	21.5

Components for treatment, cultivar, their interaction, block, and residual were estimated by restricted maximum likelihood. Several components were estimated at the boundary (zero): cultivar and the treatment × cultivar interaction for both chlorophyll traits, and block for leaf length/width. Treatment has only four levels, so its component is indicative; the fixed-effect tests in **Table 3** remain primary.

Because independent spectroradiometric scans were not recorded, *φ* is an estimate; the channel cross-sections were calibrated to reproduce Sager-based PSS values reported for fully specified recipes by Lanoue et al. (2022), which were recovered to within 0.001 root mean square (RMS). For each treatment, we integrated the Pfr-deficit (*φ*ref − *φ*, where *φ*ref is the far-red-free reference state) over an early-leaf competence window (days 0–12), motivated by evidence that spinach registers photoperiodic floral signals during early transplant development (Chun et al., 2000). Tray-level bolting counts and fresh weight were then related to the early-window Pfr-deficit and, separately, to cumulative far-red dose; the non-nested deficit and dose models were compared using Akaike Information Criterion (AIC). A 2,000-draw cross-section sensitivity analysis confirmed that the treatment grouping was invariant to coefficient choice (100% of draws). Because the early-window deficit took two distinct values, the cultivar reaction norms are two-level contrasts.

Because each timing treatment occupied a single growth tray, propagation trays within a treatment are sub-samples with respect to the treatment main effect. As a sensitivity analysis, treatment effects were therefore also tested against the treatment × cultivar mean square, that is, with cultivars acting as blocks (3 and 15 degrees of freedom; **Supplementary Table S9**). Seven of eight traits retained significance at *p* < 0.05 under this conservative test; only dry weight did not, and the treatment × cultivar interaction for dry weight was unaffected.

## Results

### Flowering incidence

Bolting incidence showed a significant treatment × cultivar interaction (beta-binomial GLMM, likelihood-ratio *χ*^2^(15) = 38.2, *p* < 0.001; **Figure 2**). Across cultivars, EarlyFR (73%) and FullFR (71%) increased flowering relative to control (50%) and LateFR (47%). The cultivars were separated into two broad groups. Amador, Harp, and Rubino flowered heavily in all treatments, including control (69%–96%), whereas Renegade, Responder, and Santa Cruz showed low flowering under control and LateFR (8%–24%) but increased sharply under EarlyFR and FullFR. One combination, Harp under LateFR, reached 100% flowering. LateFR produced the lowest flowering incidence in three of six cultivars (Santa Cruz, Responder, and Amador) and matched control in Renegade. Bolting responses were consistent on the odds scale: early far-red increased the odds of bolting approximately five- to eightfold in the four cultivars not close to the upper limit (Renegade 8.4, Responder 5.5, Rubino 5.1, and Santa Cruz 4.7), so the much larger differences in absolute incidence between cultivars reflect differences in baseline propensity rather than in sensitivity to far-red. Excluding Harp, the cultivar in which one treatment reached 100% bolting, the interaction was no longer significant (*χ*^2^(12) = 16.0, *p* = 0.19), whereas excluding any other cultivar left it significant (*p* ≤ 0.003; **Supplementary Table S10**).

**Figure 2:**
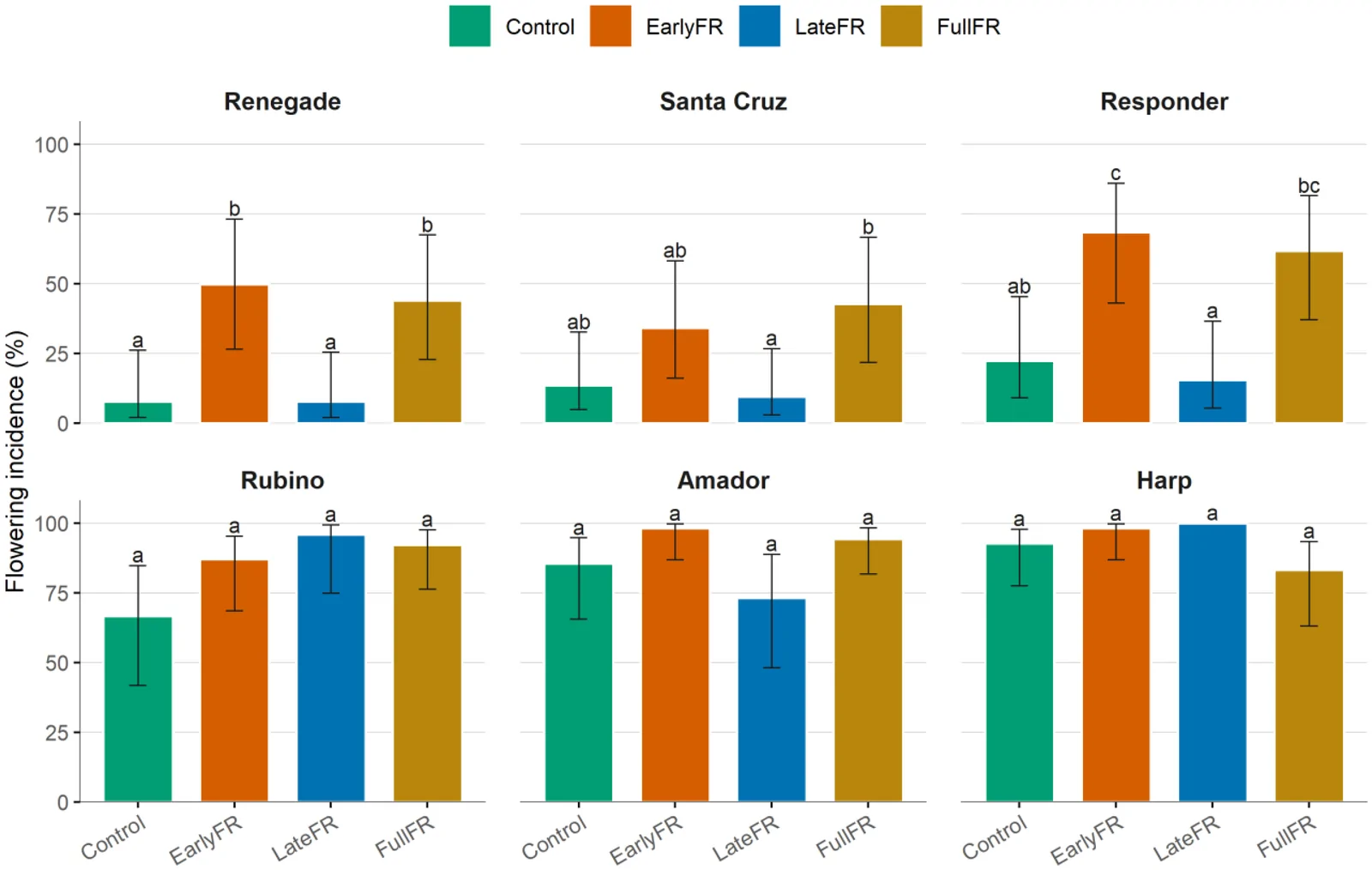
Flowering incidence (%) at harvest across far-red timing treatments, faceted by cultivar. Flowering incidence is the number of flowering or bolting plants divided by the number of plants scored in each propagation tray at harvest. Bars are estimated probabilities from the beta-binomial generalized linear model described under “Statistical analysis”, with error bars showing 95% confidence intervals. Letters indicate Sidak-adjusted significance groups among treatments within each cultivar; groups are not comparable between cultivars. Cultivars are ordered by flowering incidence under the control treatment, so the three cultivars in the upper row are those with low baseline bolting and the three in the lower row are those that bolted readily under all treatments. One bar carries no confidence interval: Harp under LateFR reached 100% bolting, so its estimate lies at the parameter boundary and no interval is identifiable.

### Marketable yield and dry shoot biomass

Fresh weight differed significantly among treatments (*F*(3, 72) = 9.42, *p* < 0.001) and cultivars (*F*(5, 24) = 8.56, *p* < 0.001), whereas the treatment × cultivar interaction was weak and not significant (*F*(15, 72) = 1.37, *p* = 0.18). Treatment marginal means separated under Tukey comparison, with LateFR producing the greatest fresh weight (244 g, group c) and EarlyFR the lowest (158 g, group a); control (224 g, group bc) and FullFR (192 g, group ab) were intermediate (**Figure 3a**). When flowering proportion was included as a covariate, both flowering (*F*(1, 93.6) = 5.60, *p* = 0.020) and treatment (*F*(3, 75.2) = 11.80, *p* < 0.001) remained significant. The yield advantage of LateFR over EarlyFR and FullFR was, therefore, not explained solely by differences in flowering.

**Figure 3:**
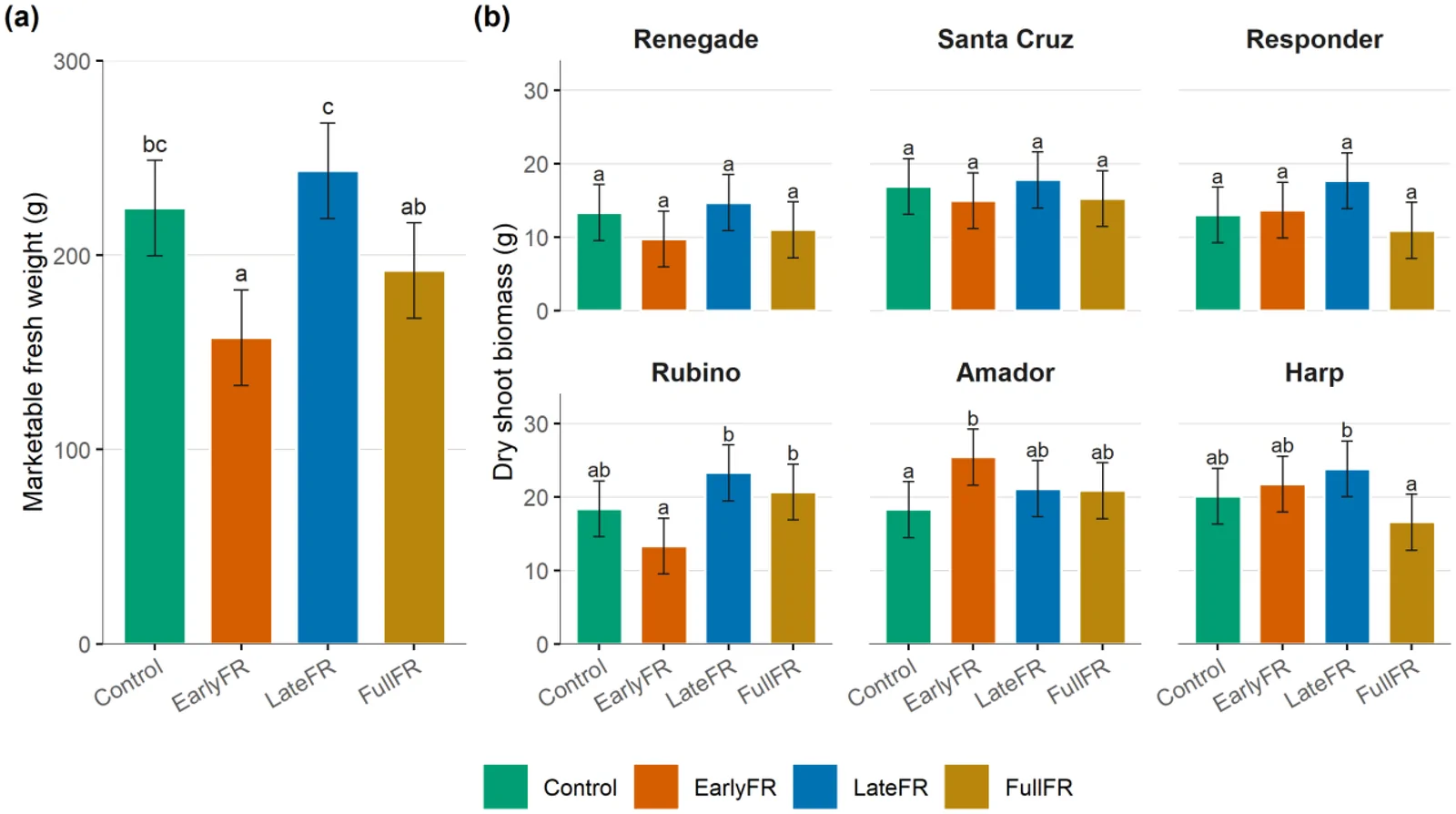
Marketable fresh weight and dry shoot biomass under the four far-red timing treatments. **(a)** Marketable fresh weight by treatment, shown as estimated marginal means with 95% confidence intervals from the tray-level linear mixed model described under “Statistical analysis”. Letters indicate Tukey-adjusted significance groups. Cultivars are pooled for this trait because the treatment × cultivar interaction was not significant (*F*(15, 72) = 1.37, *p* = 0.18). **(b)** Dry shoot biomass by treatment for each cultivar separately since the treatment × cultivar interaction was significant for this trait (*F*(15, 72) = 1.86, *p* = 0.043) and pooling would obscure it. Values are estimated marginal means with 95% confidence intervals from the same model; letters indicate Tukey-adjusted significance groups among treatments within each cultivar and are not comparable between cultivars. Cultivars are shown in the same order as **Figure 2**, that is, by flowering incidence under the control treatment, such that the upper row holds the three cultivars with low baseline bolting and the lower row the three that bolted readily under all treatments.

Dry weight showed a significant treatment × cultivar interaction (*F*(15, 72) = 1.86, *p* = 0.043): the response to far-red timing differed in direction between cultivars, with EarlyFR giving the lowest dry weight in Renegade and Rubino but the highest in Amador (**Figure 3b**).

### Cultivar differences in the magnitude of the timing response

Although the treatment × cultivar interaction for fresh weight was not statistically significant (*F*(15, 72) = 1.37, *p* = 0.18), the magnitude of the yield response to far-red timing differed markedly between cultivars (**Figure 4**). Expressed as the fresh-weight gain from switching far-red from early to late application, the recovery ranged from 0% in Amador to 37% in Harp, 67% in Responder, 91% in Santa Cruz, and 106%–107% in Renegade and Rubino.

**Figure 4:**
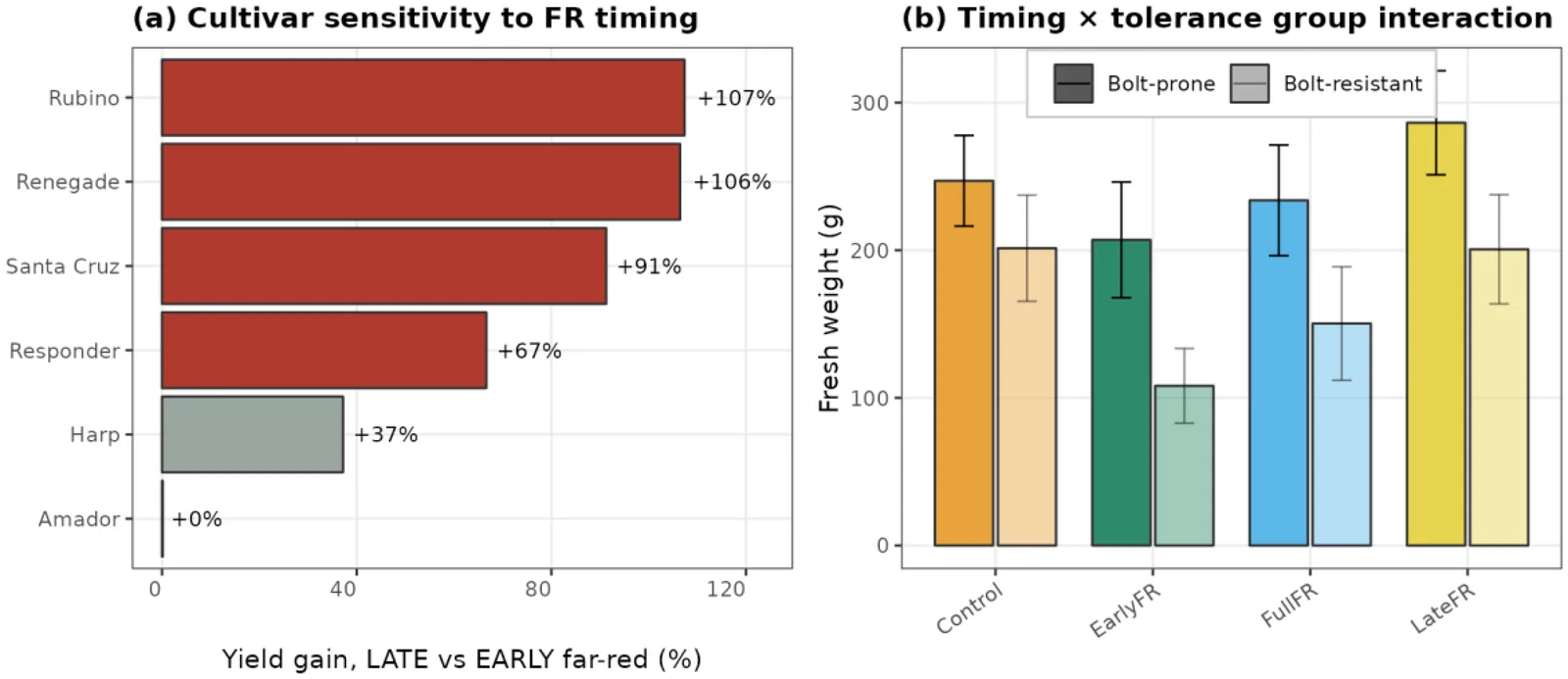
Genotype × environment × management interaction. **(a)** Fresh-weight gain (%) from switching far-red from early to late application, by cultivar. The highlighted bars indicate timing gains greater than 50%. **(b)** Mean fresh weight by treatment for bolt-prone and bolt-resistant cultivar groups.

When cultivars were grouped by bolting behavior, the timing response was most pronounced in the bolt-resistant group. These cultivars maintained low flowering under control and LateFR but lost substantial fresh weight under EarlyFR, consistent with early FR inducing bolting in genotypes that otherwise avoided it. By contrast, the bolt-prone group flowered heavily across treatments and showed a smaller relative timing response.

### Far-red timing acts through early-window phytochrome state, not dose

The treatments dissociated far-red timing from far-red dose. Integrating the estimated Pfr-deficit over the early-leaf window rendered EarlyFR and FullFR equivalent (≈0.37 *φ* · *d*) and LateFR equivalent to the control (≈0 *φ* · *d*) despite a near-threefold range in cumulative far-red dose (EarlyFR 13.5, LateFR 21.3, and FullFR 35.9 mol m^–2^; **Figures 5A, B**). Bolting followed early-window phytochrome state rather than dose: refitted at tray level, the early-window Pfr-deficit model was preferred over cumulative far-red dose for both bolting (ΔAIC = 33.2) and marketable fresh weight (ΔAIC = 22.1), and for bolting it fitted marginally better than a saturated model treating the four light regimes as unconstrained levels despite using 12 fewer parameters (ΔAIC = 1.8; **Supplementary Table S5**). Because both predictors are reparameterizations of the same four treatments, this comparison is descriptive rather than a formal test.

**Figure 5:**
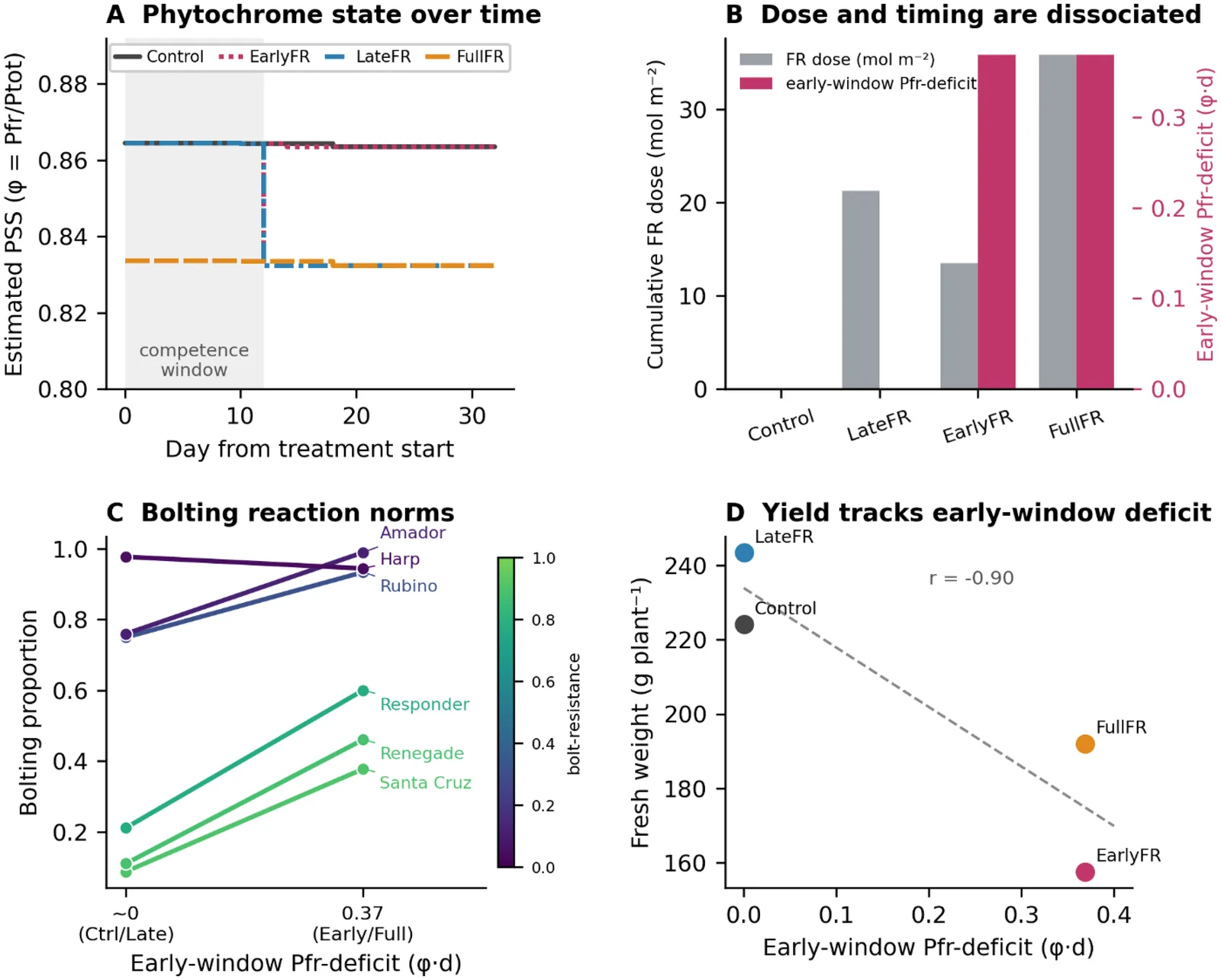
Far-red timing acts through early-window phytochrome state, not dose. **(A)** Estimated phytochrome photostationary state (*φ*) over the production cycle for each treatment; shading marks the assumed early-leaf competence window (days 0–12). **(B)** Cumulative far-red dose and integrated early-window Pfr-deficit per treatment, showing that EarlyFR carries the least dose yet the same early-window deficit as FullFR. **(C)** Cultivar reaction norms for bolting against the early-window Pfr-deficit (a two-level contrast), colored by bolting tolerance. **(D)** Treatment-mean fresh weight per propagation tray against early-window Pfr-deficit (*r* = −0.90). *φ* values are estimates calibrated to published Sager-based PSS (Lanoue et al., 2022).

Marketable yield showed the same signature: across treatment means, fresh weight was strongly and negatively associated with the early-window deficit (*r* = −0.90) but essentially unrelated to far-red dose (*r* = −0.14), with EarlyFR yielding least despite receiving the least far-red (**Figures 5C, D**).

The bolting response to the early-window deficit was strongly cultivar-dependent (deficit × cultivar interaction, Wald *χ*^2^ = 69.4, df = 5, *p* = 1.4 × 10^–13^; deficit main effect *χ*^2^ = 50.3, *p* = 1.3 × 10^–12^). Under control and LateFR, the cultivars spanned the full range from near-obligate bolting (Harp, Amador) to strong tolerance (Santa Cruz, Renegade; control bolting ≈ 0.10–0.12). Early-window far-red largely abolished this contrast: in the bolt-tolerant cultivars, bolting rose from a mean of 0.14 without early-window far-red to 0.48 with it, whereas the bolt-prone cultivars were already near ceiling and changed little (**Figure 5C**). The effect is, therefore, best described as an erosion of the bolting tolerance that distinguishes resistant genotypes rather than as a uniform sensitivity gradient; because bolt-prone cultivars approach the response ceiling, the per-cultivar sensitivity does not scale monotonically with tolerance on the latent scale (Spearman *ρ* = 0.37, *p* = 0.50, *n* = 6).

### Plant height

Height was analyzed across T1 (day 17) and T2 (harvest) at tray level using a time × treatment × cultivar model. Time explained the largest component of variation, as expected for a growth trait (*F*(1, 96) = 1,042.2, *p* < 0.001). Treatment (*F*(3, 72) = 16.77, *p* < 0.001), cultivar (*F*(5, 24) = 68.8, *p* < 0.001), time × cultivar (*F*(5, 96) = 95.7, *p* < 0.001), and time × treatment (*F*(3, 96) = 4.46, *p* = 0.006) were significant, whereas the treatment × cultivar interaction (*F*(15, 72) = 1.26, *p* = 0.25) and the three-way interaction (*F*(15, 96) = 0.81, *p* = 0.66) were not. At T1, treatments did not differ within cultivars except Rubino (**Figure 1**, upper row). By T2, LateFR and FullFR produced the tallest plants, averaging approximately 33% and 32% above control, respectively, and the bolt-prone cultivars were tallest overall.

### Leaf morphology

Leaf length differed among treatments (*F*(3, 72) = 20.42, *p* < 0.001), with control and LateFR producing longer leaves than EarlyFR and FullFR. Cultivar was also significant (*F*(5, 24) = 16.54, *p* < 0.001), but the treatment × cultivar interaction was not (*F*(15, 72) = 1.52, *p* = 0.12). Leaf width showed a similar response (treatment: *F*(3, 72) = 17.87, *p* < 0.001; cultivar: *F*(5, 24) = 25.71, *p* < 0.001; interaction: *F*(15, 72) = 1.78, *p* = 0.054), with the widest leaves under control. The leaf length-to-width ratio was increased only under LateFR (treatment: *F*(3, 72) = 9.06, *p* < 0.001; cultivar: *F*(5, 24) = 100.99, *p* < 0.001; interaction: *F*(15, 72) = 1.31, *p* = 0.22; **Figure 6**). This pattern is consistent with shade-avoidance elongation, particularly under late FR. Because the leaf and the petiole were measured together, the ratio reflects combined leaf-and-petiole elongation rather than lamina shape alone.

**Figure 6:**
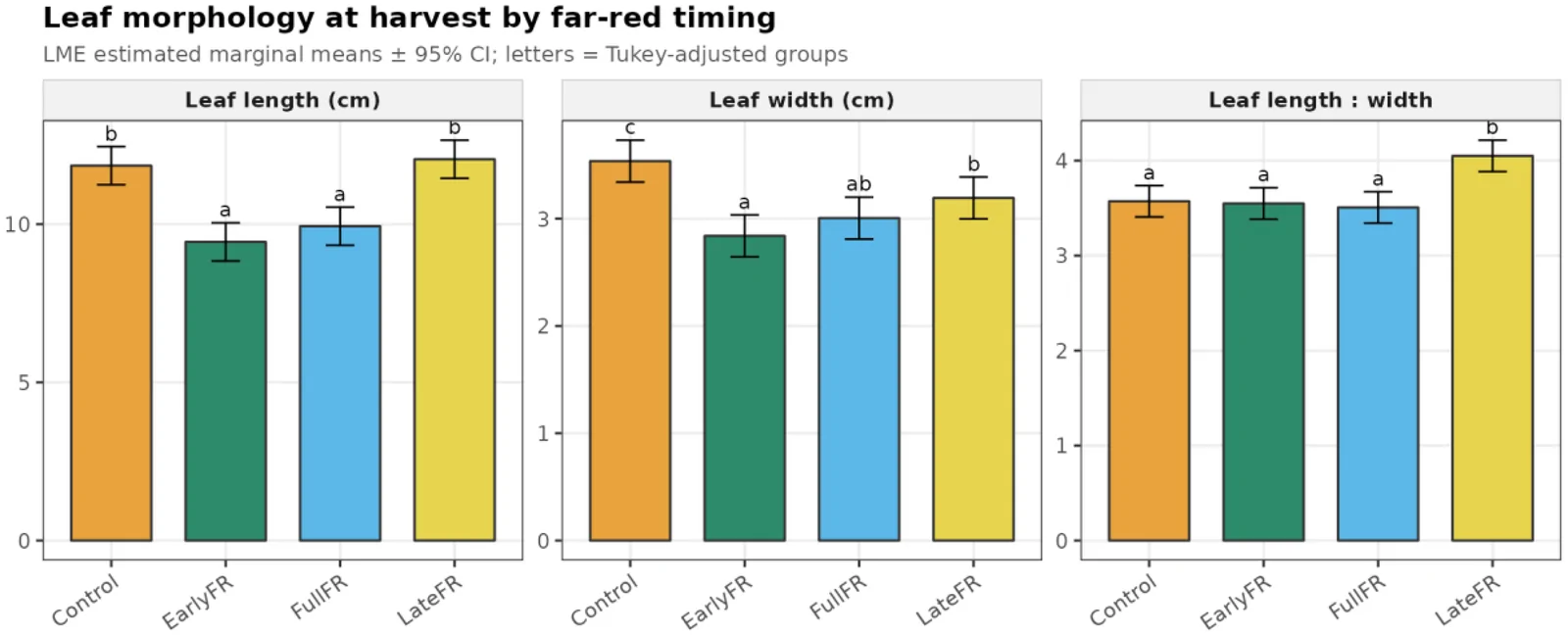
Leaf morphology at harvest (T2): leaf length (left), leaf width (centre), and leaf length-to-width ratio (right) by far-red timing. Values are LME estimated marginal means ± 95% CI; letters indicate Tukey-adjusted groups.

### Chlorophyll

Total chlorophyll differed among treatments (*F*(3, 72) = 17.3, *p* < 0.001), with no significant cultivar effect (*p* = 0.68) or treatment × cultivar interaction (*p* = 0.35). EarlyFR produced the highest total chlorophyll and was significantly greater than the other treatments when treatment effects were isolated (**Figure 7**, left). Chlorophyll a/b ratio also differed by treatment (*F*(3, 72) = 16.8, *p* < 0.001) but not by cultivar. Control had the highest a/b ratio and EarlyFR the lowest, with FullFR and LateFR being intermediate (**Figure 7**, right). The reduced a/b ratio under EarlyFR indicates relative enrichment of chlorophyll b, consistent with a shade response.

**Figure 7:**
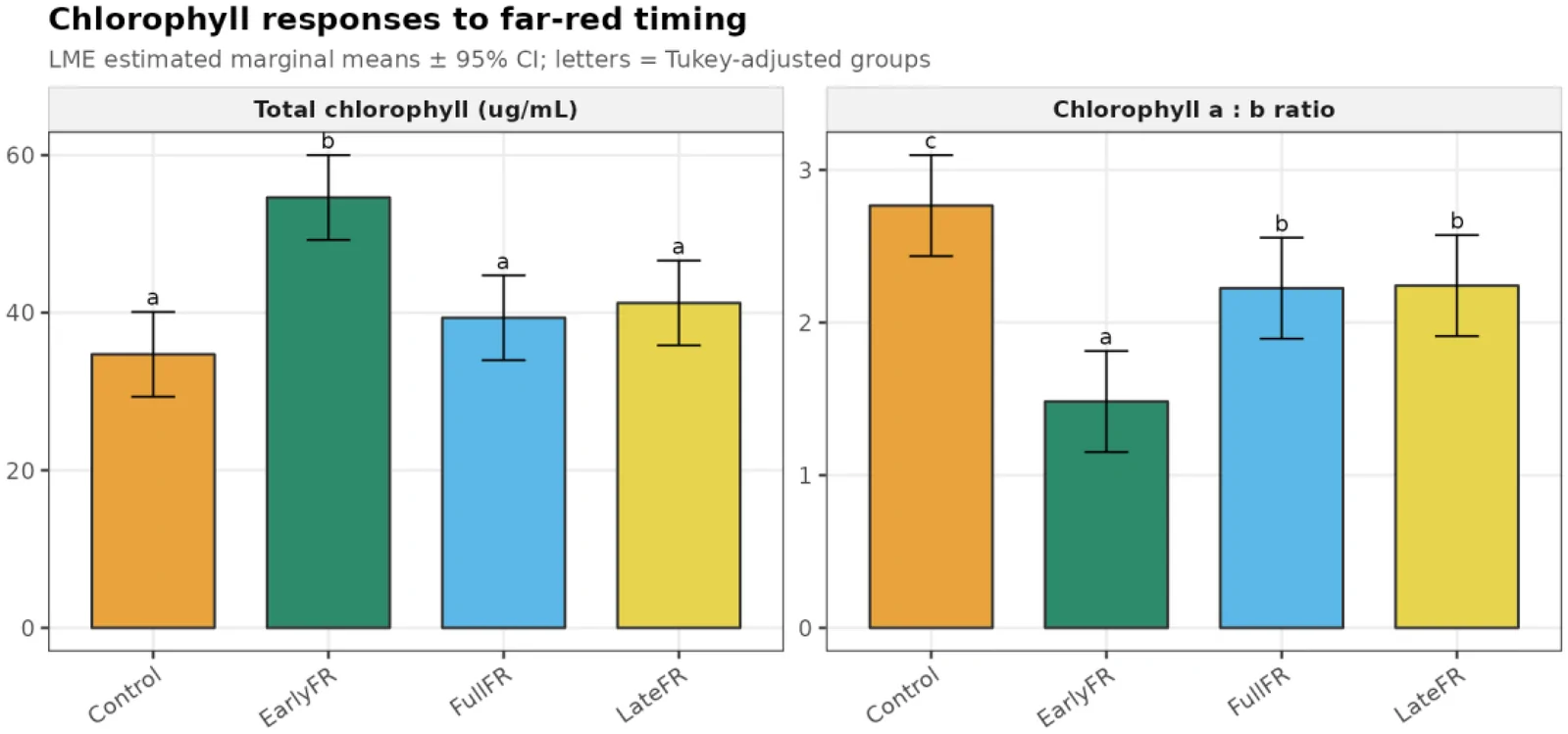
Total chlorophyll (left) and chlorophyll a/b ratio (right) by treatment. Values are LME estimated marginal means ± 95% CI; letters indicate Tukey groups.

To indicate how strongly each factor shaped the measured responses, the explained variation was partitioned among treatment, cultivar, and their interaction (**Table 4**).

Cultivar accounted for the largest single share of variance in most traits, including plant height (81%), the leaf length-to-width ratio (75%), leaf width (63%), flowering incidence (62%), leaf length (36%), and dry weight (34%), whereas far-red timing dominated only the two chlorophyll traits (34%–35%, with cultivar variance estimated at zero). Marketable fresh weight was the most evenly partitioned trait (cultivar 22%, treatment 15%, interaction 5%, and residual 54%). The treatment × cultivar interaction was a minority component in every trait, from 0.3% for height to 8.8% for dry weight, and reached significance only for dry weight and bolting incidence (**Table 3**). Its agronomic weight for cultivar-specific light management is therefore not reflected in its share of variance alone, and larger trials would allow this genotype × environment × management component to be estimated with greater precision. Fixed-effect tests for every trait are consolidated in **Table 3** and the corresponding variance components in **Table 4**.

## Discussion

### Does far-red timing affect spinach production?

This study demonstrates that the developmental window in which supplemental FR is applied, rather than its cumulative dose, governs the agronomic outcome in spinach.

### Why timing rather than dose

LateFR delivered more total far-red than EarlyFR (21.3 vs. 13.5 mol m^–2^) yet patterned with the unsupplemented control. LateFR matched or slightly exceeded control in marketable fresh weight, increased leaf length-to-width ratio, and did not increase bolting whereas EarlyFR and FullFR reduced yield and increased flowering. These findings shift the interpretation of FR from a fixed spectral component to a stage-specific management factor. They may also help reconcile our results with those of Skabelund et al. (2025), who found no morphological response to FR fraction in spinach. If FR sensitivity is developmentally gated, experiments that vary FR fraction while holding the exposure window constant may fail to detect responses that emerge when timing is varied directly.

### Why cultivars differ in their response

Early FR increased flowering, consistent with the role of low R/FR as a signal of neighbor proximity during early development. Under such conditions, shade-avoidance pathways can promote stem elongation and earlier reproductive transition before canopy closure (Franklin and Whitelam, 2005; Casal, 2012; Tan et al., 2022). Continuous FR maintained this signal and also increased flowering. In contrast, plants exposed to FR only later in development appeared to respond primarily through architectural adjustment, including greater height and altered leaf proportions, rather than through a comparable increase in reproductive commitment. The low blue-to-red background may have further favored PIF-mediated elongation and flowering under FR treatments (Casal, 2012; Park and Runkle, 2019). These results are consistent with the wider literature on FR and flowering but add a temporal component that has been little explored in spinach. They should also be interpreted in relation to photoperiod and temperature, both of which strongly influence spinach flowering: the 12-h photoperiod and 24 °C temperature used here are close to thresholds associated with reproductive transition in susceptible genotypes (Chun et al., 2000; Isaza et al., 2025), and phytochrome-B responses can be temperature-sensitive (Halliday et al., 2003).

Fresh weight was inversely related to flowering response. Bolting redirects growth toward stem and reproductive structures and away from harvestable leaves, reducing both yield and potential photosynthetic return (de Wit et al., 2018; Amini et al., 2025). The largest timing effects occurred in bolt-resistant cultivars because these genotypes flowered little under control and LateFR but were induced to bolt by EarlyFR. In contrast, bolt-prone cultivars flowered under most conditions and were therefore less responsive to changes in timing. Because biomass was recorded as total aerial mass, this study could not partition leaf, stem, and flower biomass directly. Future experiments should include organ-level biomass partitioning to quantify allocation shifts. Nevertheless, the practical result is clear: FR applied after early canopy establishment avoids the yield penalty of early FR, and its contribution to photosynthetic efficiency may reduce lighting requirements when timed appropriately (Zhen and Bugbee, 2020; Farhangi et al., 2025).

### Morphological and physiological correlates

The increase in leaf length-to-width ratio under LateFR, is consistent with a shade-avoidance elongation response mediated by phytochrome-B and downstream SAR regulators (Galvão et al., 2019; Paik and Huq, 2019). Because leaf and petiole were measured together, the present data cannot distinguish lamina expansion from petiole elongation and, therefore, cannot directly test specific-leaf-area effects reported in other spinach studies (Utasi et al., 2019; Skabelund et al., 2025). The chlorophyll response was less typical. EarlyFR increased total chlorophyll and reduced chlorophyll a/b ratio, whereas FR often reduces chlorophyll content in other species. One possible explanation is that early FR promoted the development of chlorophyll-b-rich light-harvesting antennae while maintaining chlorophyll a, leading to a higher total chlorophyll signal (Voitsekhovskaja and Tyutereva, 2015; Chen et al., 2021). Continuous FR may have attenuated or desensitized this response. Because chlorophyll was measured on standard leaf samples rather than per unit mass, these results should be interpreted as relative differences. Comparable far-red-driven elongation responses have recently been shown to differ markedly among genotypes in other crops; in quinoa, for example, supplemental far-red increased stem elongation in all genotypes tested but to differing degrees (Gordillo-Romero et al., 2026).

### Implications for cultivar choice and light management

Ranking the treatments on marketable yield and flowering liability together places LateFR first and EarlyFR last, with LateFR not differing from control but differing significantly from EarlyFR and FullFR (**Figure 8a**). A composite cultivar ranking based on high yield, low flowering, and high chlorophyll identified Santa Cruz as the strongest overall cultivar. Although Santa Cruz ranked fourth for raw fresh weight, it combined low flowering incidence, high chlorophyll, and compact growth (**Figure 8b**). High-yielding but bolt-prone cultivars, including Amador and Rubino, ranked lower once flowering liability was included.

**Figure 8:**
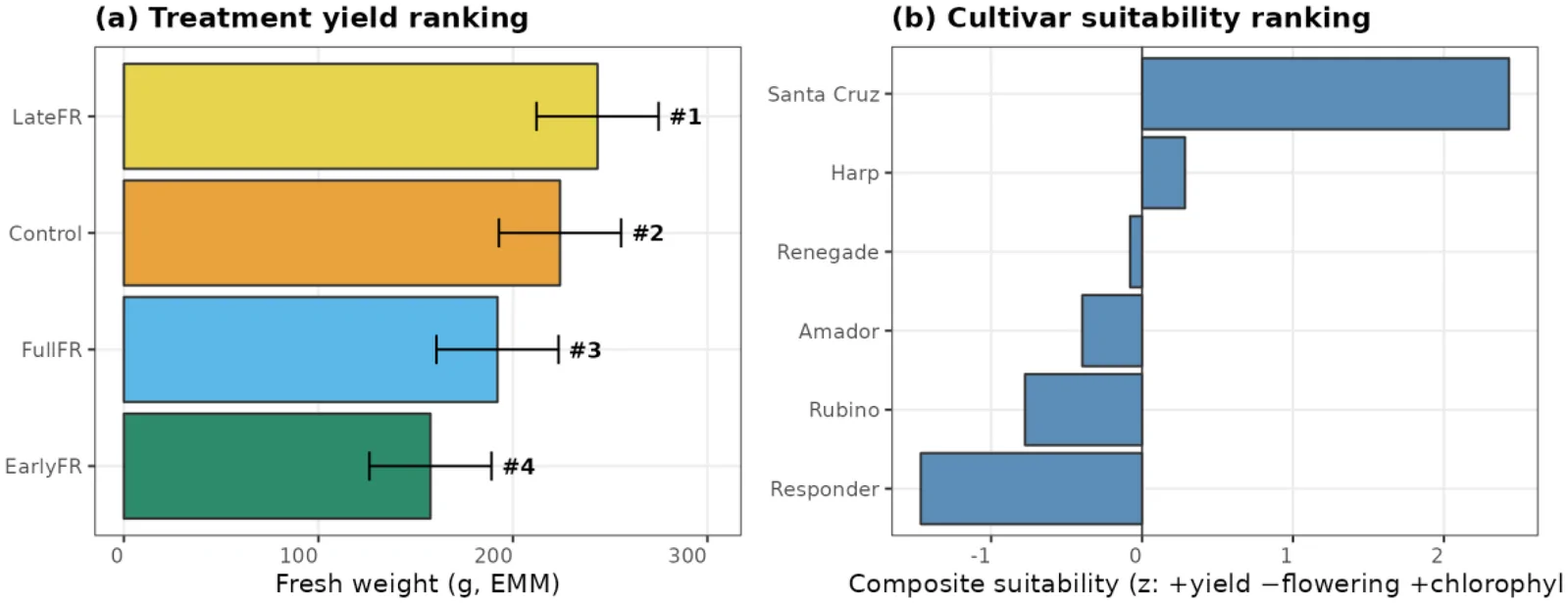
Suitability for vertical-farm production. **(a)** Treatment ranking by marketable fresh weight (estimated marginal means; #1 = best). **(b)** Composite cultivar suitability based on standardized positive yield, negative flowering, and positive chlorophyll scores; Santa Cruz ranked highest overall.

The results together define a G × E × M interaction in which the effect of FR enrichment depends on both application timing and cultivar bolting tolerance. For vertical-farm production, two management implications follow. First, supplemental FR should be applied after the early growth stage rather than throughout production or during early establishment. Second, cultivar choice should be made in relation to FR timing. Santa Cruz ranked highest overall because it combined low flowering, high chlorophyll, and compact growth, whereas high-fresh-weight but bolt-prone cultivars ranked lower when flowering was penalized. The same principle may be relevant beyond vertical farming because cultivar selection and managed light environments could help extend harvest windows under increasingly variable production conditions. Genotype-dependent responses to light quality of this kind are increasingly reported across crops, including a recent demonstration in quinoa that supplemental far-red, red, and blue light altered growth and stress responses in a strongly genotype-specific manner (Gordillo-Romero et al., 2026), reinforcing the value of evaluating spectral management within a genotype-aware framework.

### From management response to genetic gain

The genotype × management interaction documented above (see “Cultivar differences in the magnitude of the timing response”; **Table 4**) has a direct quantitative-genetic reading. Within the breeder’s equation: (Falconer and Mackay, 1996), the response to selection for marketable yield depends on the genetic variance and heritability that are actually expressed, and both are conditioned by the management regime. Early far-red compressed the cultivar panel toward bolting and yield loss, whereas late far-red raised the mean and separated cultivars according to their intrinsic bolting tolerance; management, therefore, determines which genetic differences are visible to selection. Cultivar means were highly repeatable across the four far-red regimes, with entry-mean repeatability (Holland et al., 2003): of 0.88 for marketable fresh weight and 0.93 to 0.99 for the other agronomic and architectural traits (**Supplementary Table S6**), so cultivar ranking under a fixed light regime is estimated with useful precision even in a panel of this size. Optimizing genotype and management jointly—rather than fixing a light recipe and then choosing a cultivar or the reverse—is, thus, expected to increase the achievable rate of genetic gain—the expected response to selection per unit time (ΔG per year) rather than per generation—which is the central argument for embedding G × E × M within predictive-breeding frameworks (Cooper et al., 2023; Powell et al., 2026). CEVFs are unusually well suited to this approach. Because the trial environment is the production environment, the alignment between the evaluation environment and the target population of environments approaches unity, removing much of the genotype-by-environment uncertainty that constrains gain in field programs (Powell et al., 2026), and because growth is rapid and season-independent, several generations can be advanced per year rather than the single annual cycle typical of field production. Treating far-red timing as a heritability-modifying management variable and selecting cultivars within the chosen regime, therefore, offers a tractable route to accelerate the improvement of indoor leafy crops. We did not estimate narrow-sense heritability or genetic gain directly because the six cultivars were a fixed commercial panel rather than a segregating population; the projections in **Supplementary Figure S1** are intended as a quantitative framing of this opportunity and a guide to the larger, pedigreed trials that would be required to realize it.

### Limitations

Several limitations qualify these conclusions. First, height at T2 partly reflects bolting and should not be interpreted as an independent vegetative-growth trait. Second, total aerial biomass was measured without separating leaf, stem, and reproductive tissues, limiting inference about allocation. Third, chlorophyll was extracted from two leaves per tray and expressed relative to a common protocol rather than per unit leaf mass, so the chlorophyll results should be treated as indicative; sampling leaf discs from a larger number of leaves per tray would give a more robust estimate in future work. Fourth, FR was supplied additively; therefore, total photon flux differed between FR and non-FR phases even though PAR was constant. The observed effects cannot be attributed exclusively to FR signaling independent of a small increase in total photons. We addressed this dose confound directly: because LateFR carried a larger far-red dose than EarlyFR yet produced the opposite response, the outcomes cannot be explained by cumulative photon dose, and an estimated phytochrome-state metric outperformed a dose model (see “Far-red timing acts through early-window phytochrome state, not dose”). The phytochrome photostationary state was nonetheless calculated from programmed light recipes rather than measured spectra (channel cross-sections calibrated to reproduce published Sager-based values; Lanoue et al., 2022), the early competence window was specified *a priori* and is partly confounded with the EarlyFR treatment, and the early-window deficit took only two distinct values, so the cultivar reaction norms represent two-level contrasts rather than continuous dose–response relationships. Spectral composition was not characterized during the experiment: the Skye sensor responds only between 400 and 700 nm, and the LI-COR LI-180 readings were taken after the growing period and used only for PPFD, so reported red to far-red ratios and phytochrome photostationary states are calculated from programmed channel outputs rather than measured. Measured photosynthetic photon flux density exceeded the programmed set point by 20%–30%; the manufacturer defines the programmed value as a nominal spatial mean across the entire growing area, and our sampling did not weigh the low-irradiance perimeter in proportion to its area, which may account for part of this difference. Fifth, because spectrally distinct recipes cannot be applied within a single illuminated growing space, each treatment occupied one growth tray, and treatment is not separable from growing position; cultivar effects and the cultivar × treatment interaction are replicated and randomized within every treatment and are unaffected, but treatment main effects are conditional on the four growth trays providing equivalent environments, which the light and climate records support. Finally, replication was modest (five trays per cultivar per treatment), consistent with the non-significant fresh-weight interaction. These limitations do not alter the main timing result but identify priorities for follow-up experiments.

## Conclusion

Far-red timing altered the developmental and agronomic response of spinach grown in a controlled-environment vertical-farm system. Late FR avoided the yield and bolting penalties of early FR and increased leaf length-to-width ratio, whereas early FR increased flowering and reduced yield. Early FR also increased total chlorophyll and reduced chlorophyll a/b ratio, indicating that physiological as well as morphological responses depended on timing. The magnitude of these effects varied among cultivars and was greatest in cultivars with lower baseline bolting. We conclude that supplemental FR should be applied after early growth and that cultivar selection and FR timing should be optimized jointly as a G × E × M decision. Framing these timing effects within the breeder’s equation further suggests that vertical farms, in which the trial and production environments coincide and breeding generation intervals can be short, are a favorable setting in which to optimize genotype and far-red management jointly for genetic gain (Powell et al., 2026). Future work should partition biomass by organ, quantify chlorophyll per unit mass, equalize total photon flux, and test FR timing under contrasting photoperiod and temperature regimes.

## Data Availability

The datasets and R scripts supporting this study are openly available in Zenodo at https://doi.org/10.5281/zenodo.22280237. Further inquiries can be directed to the corresponding author.

## Supplementary material

Supplementary Table 1 Phytochrome photostationary state (PSS) predictors. Experimental treatment details including Far-red (FR) window timing, cumulative FR dose (mol m^–2^), and associated PSS values ( $\phi_{FR\_on}$, $\phi_{FR\_off}$) and Early-window Pfr-deficit ( $\phi_{d}$) metrics.

Supplementary Table 2 PSS calibration anchors. Validation data comparing target PSS values derived from Sager et al. benchmarks against fitted PSS values across various source treatments.

Supplementary Table 3 Spectral cross-sections and channel-specific PSS. Summary of spectral sensitivity parameters ( $\sigma_{r}$, $\sigma_{fr}$) and calculated PSS for the blue, green, red, and far-red light channels.

Supplementary Table 4 Cultivar bolting resistance and flowering incidence. Bolting resistance scores and flowering incidence (%) for six spinach cultivars (Amador, Harp, Renegade, Responder, Rubino, Santa Cruz) across four light treatments: Control, LateFR, EarlyFR, and FullFR.

Supplementary Table 5 Comparison of two treatment-level predictors of the far-red response, fitted on tray-level data (n = 120). Bolting was modelled with a beta-binomial GLMM (logit link, block as a random effect) on the counts of flowering plants out of plants scored; marketable fresh weight with a linear mixed model (block random), fitted by maximum likelihood. The early-window Pfr-deficit takes two distinct values across the four treatments (0.0004 and 0.369 φ·d) and cumulative far-red dose takes four (0, 13.5, 21.3 and 35.9 mol m^–2^). The saturated model, in which treatment enters as an unconstrained four-level factor, is included as a reference for how much of the treatment signal each mechanistic predictor recovers. ΔAIC is relative to the best model for each response. Because both predictors are reparameterizations of the same four treatments, this is a descriptive model comparison and not a hypothesis test.

SUPPLEMENTARY TABLE 6 Entry-mean repeatability of cultivar differences. Repeatability is reported in place of broad-sense heritability because cultivar was represented by a fixed panel of six commercial varieties rather than a segregating population. Values are derived from the cultivar F-ratio of the tray-level split-plot model as w = 1 − 1/F and are an upper bound on what would be realised when selecting on single plots.

SUPPLEMENTARY TABLE 7 Programmed light quality per growth phase. Per-channel photon flux, PPFD, total and yield photon flux density, R:FR and daily light integral for each treatment phase, computed from the programmed LED recipe.

SUPPLEMENTARY TABLE 8 Cultivar-to-position assignment. The position of each cultivar within the 6 × 5 grid of propagation trays, randomised once and used identically in all four growth trays. Grid columns 1–5 each contain all six cultivars exactly once and were treated as complete blocks.

SUPPLEMENTARY TABLE 9 Conservative test of treatment effects. Because each timing treatment occupied a single growth tray, treatment effects were also tested against the treatment × cultivar mean square, that is with cultivars acting as blocks (3 and 15 degrees of freedom). Seven of the eight traits retained significance at p < 0.05; only dry weight did not.

SUPPLEMENTARY TABLE 10 Leave-one-cultivar-out test of the bolting interaction. Likelihood-ratio test of the treatment × cultivar interaction in the beta-binomial GLMM, repeated with each cultivar removed in turn. The interaction was significant in every case except when Harp was excluded, the cultivar in which one treatment combination reached 100% bolting.

SUPPLEMENTARY TABLE 11 Measured photosynthetic photon flux density. **(a)** Summary by recipe for both instruments, with coefficients of variation, ranges and the instrument difference. **(b)** All 200 individual Skye SKP215 readings on the 10 × 5 grid spanning the growing area of each growth tray, at approximately 150 mm below the LED plane. **(c)** All LI-COR LI-180 readings by recipe and position; one LateFR reading is flagged as anomalous and excluded from the summary. Measurements were made after the growing period with the tower running the programmed experimental recipes.

SUPPLEMENTARY FIGURE 1 Projected genetic gain under the breeder’s equation, based on the entry-mean repeatability estimates in Supplementary Table S6.

SUPPLEMENTARY FIGURE 2 Spatial distribution of measured PPFD across the growing area for each light recipe. Skye SKP215 quantum sensor, 10 × 5 grid, n = 50 per recipe, common colour scale across panels. Underlying values are given in **Supplementary Table S11**.

SUPPLEMENTARY FIGURE 3 Measured PPFD does not account for the treatment responses. **(a)** All individual measurements from both instruments by recipe, against the programmed set-point band. **(b)** Recipe mean measured PPFD, with recipes coloured by whether far-red was applied in the phase relevant to each trait.

SUPPLEMENTARY FIGURE 4 Tower climate over the experimental period, logged every 30 s by the tower management system. **(a)** Air temperature, **(b)** relative humidity, **(c)** CO2. Lines are daily means, bands the daily minimum to maximum, dashed lines the programmed set points.

SUPPLEMENTARY FIGURE 5 Experimental design: far-red timing windows across the 32-day cycle, the four growth trays within the tower, and the 6 × 5 cultivar layout within each growth tray.

SUPPLEMENTARY TEXT 1 Quantitative-genetic summary of the cultivar variation, including entry-mean repeatability estimates with bootstrap confidence intervals.
