# Supplementary material for: Far-red timing uncovers cultivar-dependent yield and bolting responses in vertical-farm spinach (Spinacia oleracea L.)
Source: Front Plant Sci. 2026 Oct 13;17:1934445. doi: 10.3389/fpls.2026.1934445 (PMC13710037; doi:10.3389/fpls.2026.1934445)
Supplement: Supplementary file 1 [file DataSheet1.docx]

# Supplementary Material

Far-red timing, cultivar-dependent bolting and the case for breeding leafy-salad cultivars for Northern-latitude vertical farms

## Entry-mean repeatability and projected genetic gain

Because cultivar was represented by a fixed panel of six commercial varieties, we summarised the repeatability of each trait on an entry-mean basis, estimated from the cultivar F-ratios of the split-plot models as R = 1 − 1/F (Falconer and Mackay, 1996; Holland et al., 2003; Supplementary Table S6). Repeatability was high for the agronomic and architectural traits — marketable fresh weight (repeatability = 0.88), dry weight (0.93), the leaf dimensions (0.94–0.99) and plant height (0.98) — but was not estimable for the two chlorophyll traits, for which cultivar was non-significant. These values describe the among-cultivar signal expressed across the four far-red regimes and are therefore an upper bound on the heritability that would be realised when selecting on single plots; we use them to anchor, rather than to fix, the projections below. Using the breeder’s equation in its response-to-selection form (ΔG = i r σ_G), projected gain increased with heritability and selection intensity as expected (Supplementary Fig S1a). Notably, at the repeatability estimated here, simple phenotypic selection on marketable yield matched or exceeded the gain from a moderate-accuracy genomic-prediction scenario, and genomic prediction became the more efficient route only at lower repeatabilities (Supplementary Fig S1a). When the same expression was placed in its extended form (ΔG = i r_MET r_MET,TPE σ_G / L; Cooper et al., 2023), the compressed breeding generation interval achievable indoors (of the order of weeks to a few months, against a single field cycle per year) and the close correspondence between the trial and the target production environment of a vertical farm (r_MET,TPE → 1) together projected substantially higher annual gain than a comparable open-field multi-environment trial (Supplementary Fig S1b).

## Supplementary Text S1. Entry mean repeatability and projected genetic gain

Because cultivar was represented by a fixed panel of six commercial varieties rather than a segregating population, the following quantitative-genetic summary is a descriptive projection intended to frame the breeding opportunity discussed in Section 4.6, not an estimate of realisable gain. We summarised the consistency of cultivar ranking as an entry-mean repeatability, estimated as 1 − 1/F from the cultivar F-ratio of the tray-level split-plot model (Supplementary Table S6). Because the six entries are fixed commercial cultivars rather than a random sample from a segregating population, this quantity is not a broad-sense heritability and no genetic variance is estimable from it; it is used here only as an upper bound on the accuracy with which cultivar means can be ranked, to frame the breeding opportunity discussed.

Using the breeder’s equation in its response-to-selection form (ΔG = i r σ_G), projected gain increased with heritability and selection intensity (Figure S1A). At the heritabilities estimated here, simple phenotypic selection on marketable yield matched or exceeded a moderate-accuracy genomic-prediction scenario, which became the more efficient route only at lower heritabilities. In the extended form (ΔG = i r_MET r_MET,TPE σ_G / L; Cooper et al., 2023), the short breeding generation interval achievable indoors and the close correspondence between the trial and the target production environment of a vertical farm (r_MET,TPE → 1) together projected substantially higher annual gain than a comparable open-field multi-environment trial (Figure S1B). Because a vertical farm’s trial environment is also its production environment, the alignment between evaluation and target environments approaches unity, removing much of the genotype-by-environment uncertainty that constrains gain in field programmes (Powell et al., 2026).

### Supplementary Table S6. Cultivar effects and entry-mean repeatability for each trait.

F-ratios are Type III tests of cultivar against the whole-plot error term of the split-plot model, fitted on tray-level means (n = 120 trays; Kenward–Roger denominator degrees of freedom). Repeatability on an entry-mean basis was calculated as 1 − 1/F, where each cultivar mean is taken over 20 trays (four light treatments × five blocks). It expresses the proportion of variation among cultivar means that is attributable to consistent cultivar differences under these growing conditions, and is not a broad-sense heritability: the six entries are fixed commercial cultivars rather than a random sample from a segregating population, so no genetic variance component is estimable. Traits for which cultivar was not significant are not estimable (n.e.).

| **Trait** | **Cultivar F (df 5, 24)** | **p** | **Entry-mean repeatability** |
| --- | --- | --- | --- |
| Marketable fresh weight | 8.56 | 9.2 × 10⁻⁵ | 0.88 |
| Dry weight | 13.70 | 2.2 × 10⁻⁶ | 0.93 |
| Plant height (T2) | 64.39 | 4.1 × 10⁻¹³ | 0.98 |
| Leaf length (T2) | 16.54 | 4.3 × 10⁻⁷ | 0.94 |
| Leaf width (T2) | 25.71 | 6.6 × 10⁻⁹ | 0.96 |
| Leaf length:width (T2) | 100.99 | 2.6 × 10⁻¹^5^ | 0.99 |
| Total chlorophyll | 0.62 | 0.684 | n.e. |
| Chlorophyll a:b | 0.75 | 0.591 | n.e. |

### Figure S1. Projected genetic gain for vertical-farm spinach under the breeder’s equation.

(A) Response to selection per cycle, in genetic standard-deviation units, versus broad-sense heritability and selection intensity for phenotypic selection (solid lines; accuracy r = √H²) and a moderate-accuracy genomic-prediction scenario (dashed, 10 % selected); the marker is the entry-mean heritability for marketable fresh weight. (B) Annual gain from the extended breeder’s equation against breeding generation interval (shaded band = plausible controlled-environment range ~60–120 d) for a vertical farm, a glasshouse and an open-field multi-environment trial. These are descriptive projections; realising them would require pedigreed populations and direct genetic-variance estimates.

### Figure S2. Spatial distribution of measured photosynthetic photon flux density across the growing area for each light recipe.

Measurements were made with a Skye SKP215 quantum sensor on a 10 × 5 grid spanning the growing area of each growth tray (n = 50 per recipe), at approximately 150 mm below the LED plane. Each panel is oriented as the tray is, with length left to right and width top to bottom; the colour scale is common to all four panels. Recipe means were 330.8 (Control), 333.0 (EarlyFR), 315.3 (FullFR) and 334.8 (LateFR) µmol m⁻² s⁻¹, with coefficients of variation of 7.5, 7.9, 11.3 and 7.7 % and ranges of 258–380, 239–394, 247–430 and 286–397 µmol m⁻² s⁻¹ respectively. Values fall towards the tray ends and outer columns in every recipe, indicating that the spatial pattern is a property of the fixture geometry rather than of any individual treatment. Full values are given in Supplementary Table S11.

### Figure S3. Measured PPFD does not account for the treatment responses.

(a) All individual measurements by recipe: closed symbols, Skye SKP215 (n = 50 per recipe); open symbols, LI-COR LI-180 (n = 9–20); horizontal bars, recipe means; shaded band, the programmed set point of 257–264 µmol m⁻² s⁻¹. **(b)** Recipe mean measured PPFD, with recipes coloured by whether far-red was applied in the developmental phase relevant to each trait. In both traits the phenotypic groups interleave along the PPFD axis rather than separating along it: the two recipes with the highest bolting incidence differ by 5.6 % in measured PPFD and span almost the entire measured range, while the two with the lowest lie between and above them, and the two recipes producing the tallest plants are the two extremes of the distribution. EarlyFR and LateFR differ by 0.5 % in measured PPFD and are identical in total far-red dose, photoperiod and daily light integral, yet fall in opposite groups for both traits.

### Figure S4. Measured tower climate overview.

Tower climate over the experimental period, logged every 30 s by the tower management system from 26 October to 16 December 2025 (approximately 145,000 records per variable). **(a)** Air temperature, **(b)** relative humidity, **(c)** CO₂ concentration. Lines are daily means, bands the daily minimum to maximum, dashed lines the programmed set points. Logged values sat below set point throughout: temperature 23.0 °C against a set point of 24 °C, relative humidity 54.5 % against 60 %, and CO₂ 748 ppm against 800 ppm. A single sensor serves the tower, so these traces describe the shared microclimate rather than individual growth trays; because all four timing treatments ran concurrently from a single sowing date, the variation shown was experienced identically by all four.

### Figure S5. Experimental design layout.

### (a) Far-red timing windows for the four treatments across the 32-day production cycle. All treatments received an identical red–green–blue background (PPFD 260–264 µmol m⁻² s⁻¹, 12 h photoperiod, DLI 11.1–11.4 mol m⁻² d⁻¹); far-red, where applied, was supplied additively at 26 µmol m⁻² s⁻¹ (10 % of PPFD) coincident with the photoperiod. (b) Each timing treatment was applied to one growth tray within a single IGS Growth Tower (model GT6). All four growth trays were operated concurrently from a single sowing date under common temperature, humidity, CO₂, nutrient and irrigation regimes. (c) Layout within every growth tray: 30 propagation trays in a 6 × 5 grid, six cultivars with five replicate trays each and approximately 40 plants per tray. Cultivar positions were randomised once and the same layout used in all four growth trays, so each cultivar occupied the same positions in every treatment; each of the five grid columns contains all six cultivars exactly once and was treated as a complete block. Cultivar codes: A, Amador; H, Harp; Rn, Renegade; Rs, Responder; Rb, Rubino; S, Santa Cruz. The full cultivar-to-position assignment is given in Supplementary Table S8. Because spectrally distinct light recipes cannot be applied within a single illuminated growing space, the timing treatments could not be replicated across growth trays. Cultivar effects and the cultivar × treatment interaction are replicated and randomised within every treatment; treatment main effects are conditional on the four growth trays providing equivalent environments, which is supported by the light measurements in Supplementary Figures S2–S3 and the climate records in Supplementary Figure S4.

### Supplementary Table S7. Programmed light quality per growth phase.

Values are programmed LED channel outputs (µmol m⁻² s⁻¹) for the common red–green–blue background, which was identical across treatments and varied only with growth phase. Channel peaks: blue ≈ 451 nm, green ≈ 522 nm, red ≈ 660 nm, far-red ≈ 731 nm. YPFD is the yield photon flux density, computed by weighting each PAR channel by McCree’s (1972) relative quantum yield at its peak wavelength (blue 0.72, green 0.83, red 0.96) and summing over 400–700 nm. No independent spectroradiometric scan was taken; values are programmed-recipe values.

**(a) Background PAR spectrum (common to all treatments)**

| Growth phase | Blue µmol (%) | Green µmol (%) | Red µmol (%) | PPFD µmol | YPFD µmol | YPF:PPF | DLI mol m⁻² d⁻¹ |
| --- | --- | --- | --- | --- | --- | --- | --- |
| Phase 1 (d 0–10) | 64 (24.9) | 8 (3.1) | 185 (72.0) | 257 | 230 | 0.896 | 11.10 |
| Phase 2 (d 10–18) | 67 (25.8) | 8 (3.1) | 185 (71.2) | 260 | 233 | 0.894 | 11.23 |
| Phase 3 (d 18–32) | 75 (28.4) | 10 (3.8) | 179 (67.8) | 264 | 234 | 0.887 | 11.40 |

**(b) Effect of the far-red supplement (26 µmol m⁻² s⁻¹, 731 nm) in FR-on phases**

| Growth phase | FR µmol (% of TPFD) | TPFD µmol | Blue % | Green % | Red % | R:FR | TPFD-DLI mol m⁻² d⁻¹ |
| --- | --- | --- | --- | --- | --- | --- | --- |
| Phase 1 (d 0–10) | 26 (9.2) | 283 | 22.6 | 2.8 | 65.4 | 7.1 | 12.23 |
| Phase 2 (d 10–18) | 26 (9.1) | 286 | 23.4 | 2.8 | 64.7 | 7.1 | 12.36 |
| Phase 3 (d 18–32) | 26 (9.0) | 290 | 25.9 | 3.4 | 61.7 | 6.9 | 12.53 |

In FR-off phases (Control throughout; EarlyFR after d 12; LateFR before d 12) no far-red channel was active, so TPFD = PPFD and the R:FR ratio is effectively infinite. Which phases carried FR in each treatment follows the treatment schedule (manuscript Table 1). Cumulative far-red dose over the cycle was 0 (Control), 13.5 (EarlyFR), 21.3 (LateFR) and 35.9 (FullFR) mol m⁻².
